# Complete Genome Sequence of EtG, the First Phage Sequenced from Erwinia tracheiphila

**DOI:** 10.1128/genomeA.00127-18

**Published:** 2018-02-22

**Authors:** Andrés Andrade-Domínguez, Roberto Kolter, Lori R. Shapiro

**Affiliations:** a Department of Microbiology and Immunobiology, Harvard Medical School, Boston, Massachusetts, USA; b Department of Applied Ecology, North Carolina State University, Raleigh, North Carolina, USA

## Abstract

Erwinia tracheiphila is the causal agent of bacterial wilt of cucurbits. Here, we report the genome sequence of the temperate phage EtG, which was isolated from an E. tracheiphila-infected cucumber plant. Phage EtG has a linear 30,413-bp double-stranded DNA genome with cohesive ends and 45 predicted open reading frames.

## GENOME ANNOUNCEMENT

Erwinia tracheiphila (family Enterobacteriaceae) is an economically important phytopathogen that infects cultivated Cucumis melo (muskmelon), Cucumis sativus (cucumber), and Cucurbita spp. (squash, pumpkin, and gourds) in temperate northeastern and midwestern North America (1). The reference genome (2) shows structural changes associated with its recent emergence into a novel ecological niche (3). This includes the absence of clustered regularly interspaced short palindromic repeat (CRISPR) loci associated with *cas* genes, as well as an extraordinarily high number of predicted prophage regions (4). The high number of predicted prophages suggests that polylysogeny may be important for E. tracheiphila pathogenicity and/or ecological interactions.

Here, we sought to isolate phages induced *in vivo* from an E. tracheiphila-infected cucumber plant. In this infected cucumber plant, we found a phage that we have named EtG. EtG is able to infect 8 of 30 E. tracheiphila environmental isolates. A strain of E. tracheiphila carrying EtG was isolated from the same plant from which we recovered the phage EtG (unpublished data), suggesting that phage EtG is a temperate phage. The genome of phage EtG was sequenced with the Illumina MiSeq platform to ∼100× coverage. The 100-bp reads were *de novo* assembled into a single contig using Velvet (5), and phage genome ends were determined through closure PCR and Sanger sequencing.

Annotation of the open reading frames was performed with the Rapid Annotations using Subsystems Technology (RAST) (6) and PHAge Search Tool (PHAST) (7) servers, which predicted 45 coding sequences (CDSs). Of these, 34 CDSs were assigned a predicted function and 9 have no assigned function. Sequence similarity searches were performed with the translation of each predicted CDS against the NCBI protein database using BLASTp (8) in order to assign putative protein functions. tRNAscan-SE (9) was used to search for tRNAs, but none were found.

Phage EtG has a linear double-stranded DNA genome of 30,413 bp, with 19 base-cohesive ends and a G+C content of 54.1%. The phage EtG genome showed complete collinearity and 66.9% overall nucleotide identity to Escherichia virus 186 (GenBank accession number NC_001317). The two subunits of the EtG terminase (EtG_02 and EtG_03) share 98.8% amino acid identity with the Escherichia virus 186 terminase subunits, suggesting a common packaging process.

The genome of phage EtG contains a protein for DNA replication (EtG_43) and an integrase gene (EtG_33). For DNA packaging, phage EtG has a packaging protein (EtG_01) and two subunits of terminase (EtG_02 and EtG_03). There were 17 genes identified for the head, baseplate, and tail morphogenesis. Other notable genes are the repressor protein CI for the maintenance of immunity (EtG_34), the regulatory protein CII, required for the establishment of lysogeny (EtG_36), and the Apl repressor (EtG_35) that down-regulates lytic transcription. The genes for host-cell lysis are spanins, holins, and a lysozyme (EtG_10). This is the first of dozens of Erwinia tracheiphila phage genomes, many of which are likely to be novel and likely to impact the host’s ecology.

### Accession number(s).

The complete genome sequence of E. tracheiphila phage EtG was deposited in GenBank under the accession number MF276773.
